# Pediatric OSA—Spectrum of the Disease and Opportunities for Personalized Interventions

**DOI:** 10.3390/jpm16050259

**Published:** 2026-05-12

**Authors:** Hui-Leng Tan, Athanasios Kaditis, David Gozal

**Affiliations:** 1 Department of Pediatric Respiratory Medicine, Royal Brompton Hospital, London SW3 6NP, UK; hui-leng.tan1@nhs.net; 2 Division of Pediatric Pulmonology, Pediatric Sleep Center, Department of Child Health, University of Missouri School of Medicine and MUHC Children’s Hospital, Columbia, MO 65201, USA; kaditia@hotmail.com; 3 Departments of Pediatrics and Biomedical Sciences, Joan C. Edwards School of Medicine, Marshall University, Huntington, WV 25755, USA

**Keywords:** primary snoring, upper airway resistance syndrome, obstructive sleep apnea, obstructive hypoventilation, machine learning, DISE, artificial intelligence, biomarkers

## Abstract

Pediatric obstructive sleep-disordered breathing encompasses a wide spectrum of diagnostic clusters, including primary snoring, upper airway resistance syndrome, mild, moderate, and severe obstructive sleep apnea, and obstructive hypoventilation. Even within these classifications, the symptomatic presentation involves a large array of variations, reflecting a wide phenotypic spectrum. Here, we aim to summarize current diagnostic criteria and explore the spectrum of disease, particularly highlighting the phenotypic variation and its potential relevance to therapeutic decisions and overall outcomes. It has become apparent that polysomnographic (PSG) indices do not correlate well with associated morbidities, even though one-night in-lab PSGs are considered the diagnostic gold standard. Novel approaches, including exploration of plasma and urine biomarkers and data-mining the physiological information embedded within the PSG, may enable the extraction of phenotypic information that can then be interpreted in conjunction with clinical data, including history, physical examination findings, risk factors, and associated disease morbidity, so that an individualized treatment plan can be optimally delineated.

## 1. Introduction

Obstructive sleep-disordered breathing (SDB) is now recognized to be one of the most common disorders of sleep in childhood. It is “a syndrome of upper airway dysfunction during sleep, characterized by snoring and/or increased respiratory effort that results from increased upper airway resistance and pharyngeal collapsibility” [1]. In the strictest definition of the condition, its polysomnographic spectrum ranges from primary snoring, through upper airways resistance syndrome to obstructive sleep apnea syndrome (OSAS), and obstructive hypoventilation, a classification that is exclusively predicated on the findings during overnight polysomnography (PSG). Based on PSG characteristics, each of these entities has now defined criteria: Primary snoring comprises habitual snoring (>3 nights a week) without apneas, hypopneas, frequent electroencephalographic (EEG) arousals, or evidence of gas exchange abnormalities. Upper airway resistance syndrome is when habitual snoring is present but combined with increased respiratory effort and work of breathing, resulting in EEG or autonomic arousals (i.e., resulting in sleep fragmentation), but no recognizable obstructive events (apnea or hypopnea) or gas exchange abnormalities. OSAS is the recurrent occurrence of either partial or complete obstruction of the upper airway during sleep (with resultant obstructive hypopneas, obstructive or mixed apneas), causing disruption of normal oxygenation, ventilation, and sleep patterns. Finally, obstructive hypoventilation is the presence of snoring and an abnormally elevated end-expiratory carbon dioxide partial pressure in the absence of recognizable obstructive respiratory events. As can be expected from a clinical syndrome, the diagnostic criteria for pediatric OSAS as per the *International Classification of Sleep Disorders*, 3rd ed., require a combination of clinical signs and abnormalities on PSG [2]. Thus, the presence of one or more of the following signs and symptoms is required:

1. Snoring;
2. Labored, paradoxical, or obstructed breathing during the child’s sleep;
3. Sleepiness, hyperactivity, behavioral problems, or learning problems.

In conjunction with abnormal PSG findings of:

1. One or more obstructive or mixed apneas, or hypopneas, per hour of sleep and/or
2. Obstructive hypoventilation is defined as at least 25% of total sleep time with hypercapnia (PETCO_2_ > 50 mm Hg) in association with one or more of the following: snoring, flattening of the inspiratory nasal pressure waveform, and paradoxical thoracoabdominal motion. Some of the literature has used a definition of ≥2% of recording time spent with PtcCO_2_ > 50 mmHg.

In the current review article, we will initially summarize current diagnostic criteria that are based on in-laboratory polysomnography, and then explore the spectrum of disease, particularly highlighting the phenotypic variation and its potential relevance to therapeutic decisions and overall outcomes. In particular, emphasis will be given to underlying endotypes, which are major contributors to the observed phenotypic variation and may facilitate the application of targeted personalized interventions, some of which are actionable and others are currently investigational.

### Classification of Children into Phenotypic Clusters Using PSG Indices Which Result in Information for Management and Prognosis

The essential role of the PSG in the diagnosis of obstructive SDB has led to the American Academy of Pediatrics’ recommendation that the gold standard diagnostic investigation is a nocturnal, in-lab PSG study [3]. A typical montage includes EEG, chin and anterior tibial EMG, bilateral electro-oculogram, pulse oximetry and pulse waveform, nasal pressure transducer, oronasal airflow thermistor, tidal or transcutaneous capnography, chest and abdominal respiratory inductance plethysmography, body position sensor, microphone, and video monitoring. Pediatric scoring criteria should be used in children up until 18 years of age, though there is the option to score older adolescents using adult criteria [4,5]. The majority of sleep laboratories score according to the American Academy of Sleep Medicine (AASM) guidelines [6]. PSGs provide objective quantitative data regarding respiratory parameters and sleep architecture, thus allowing stratification of patients according to severity cut-offs, which facilitates the tailoring of clinical management. The presence of an obstructive apnea-hypopnea index (OAHI) of >1 but equal or less than 5 events per hour of total sleep time (h TST) is usually considered as mild OSAS, while 5 < OAHI ≤ 10/h TST corresponds to moderate OSAS, and OAHI > 10/h TST as severe OSAS.

However, this classification, while simple and easy to implement, often fails to reflect the considerable phenotypic variation in obstructive SDB. For example, children with primary snoring or UARS may manifest substantial morbidity (e.g., hypertension, hyperactivity, and inattentive behaviors, etc.) while children with severe OSAS may not exhibit any detectable morbidity. To help address this issue, PSG indices other than the OAHI also need to be taken into consideration and may contribute to a certain extent to the association between PSG measures and morbid consequences [7,8]. Examples of respiratory parameters include mean oxygen saturation of hemoglobin (SpO_2_), SpO_2_ nadir, oxygen desaturation index (either 3% or 4%), time spent with SpO_2_ < 90%, mean CO_2_, peak CO_2_, % of CO_2_ > 50 mmHg, while sleep architecture indices that may also usefully contribute include arousal index (ARtotI), respiratory arousal index (RAI), spontaneous arousal index (SAI), %N1, N2, N3, and %REM sleep.

To delineate a potential categorical classification algorithm of the SDB spectrum, Spruyt et al. conducted an unbiased data-driven analysis, without a priori assumptions or pre-set cut-offs in a large community-based cohort of 1133 children aged 5- to 9-years [9]. Principal component analysis was used to identify the uniqueness of PSG-derived measures that are routinely used in clinical practice, namely AHI, apnea index (AI), obstructive apnea index, nadir SaO_2_, SAI, and RAI. These measures were data-mined to further characterize and discriminate across categorical phenotypes. A spectrum of obstructive SDB consisting of 6 clusters was identified. Future research examining the correlation between this stratification and the specific differences in end-organ morbidity or treatment outcomes will ultimately determine its clinical impact and usefulness in the evaluation of a symptomatic child being referred for assessment.

More recently, machine learning was applied to the clinical features and PSG findings of clinically referred children. This approach in a relatively small cohort indicated the presence of three major clinical clusters [10]. However, the implications of these findings have not been pursued to date. In a study involving 22 sociodemographic, anthropometric, and clinical data from 464 children (5–10 years old) from the Childhood Adenotonsillectomy Trial (CHAT) database, a cluster analysis and subsequently an explainable artificial intelligence (XAI) approach were implemented to assess phenotypic information as an OSAS resolution predictor at baseline prior to adenotonsillectomy (AT) [11]. Our findings indicated the presence of three identifiable clusters and that children aged 6 years or older who are either overweight or obese, and those with enlarged neck and waist circumference, have reduced odds of recovering from OSAS after AT. Furthermore, a novel approach predicated on subject-based SpO_2_ weighted correlation networks and modularity analysis enabled identification of clinically relevant clusters within a pediatric OSAS population that should improve clinical decision-making and patient care [12]. Using a different approach, in-depth analysis of the PSG EEG channels, particularly slow oscillations, enabled the identification of children at risk for neurobehavioral deficits [13,14].

Tauman et al. proposed a Sleep Pressure Score (SPS) as a surrogate measure for disrupted sleep homeostasis and sleep propensity [15]. They showed in a cohort of 559 children that the ARtotI and RAI had a positive correlation with the AHI [16]. In contrast, the spontaneous arousal index (SAI) showed an inverse correlation, and this inverse relationship was hypothesized to occur because compensatory mechanisms aiming to preserve sleep homeostasis and thus lead to a decline in spontaneous arousals that partially compensate for the reciprocal increase in respiratory-related arousals. An algorithm incorporating all three arousal indices was therefore proposed, whereby:

SPS = RAI/ARTtotI + (1 − SAI/ARtotI).

The proposed cut-off for “normal” SPS is 0.25, the point at which sleep homeostatic compensatory mechanisms are overcome by the underlying disorder, and at which time the presence of excessive somnolence or one of its clinical manifestations may become apparent. Indeed, children with higher SPS (>0.25) were more likely to have deficits in memory, language abilities, verbal abilities, and some visuospatial functions than children with low SPS. Importantly, the SPS was associated with deficits in neurobehavioral daytime functions, independent of respiratory disturbance and hypoxemia [7]. This observation further reinforces the concept that the information collected from the PSG by visual scoring of events may not be sufficiently predictive of the clinical phenotype and that, therefore, children with lower severity measures in the PSG may still present with significant clinical symptoms that merit clinical management, even if the PSG appears to be normal. The Pediatric Adenotonsillectomy Trial for Snoring study [17] compared adenotonsillectomy to watchful waiting in children with mild SDB. Adenotonsillectomy did not significantly improve executive function or attention at 12 months, but secondary outcomes, such as behavior, symptoms, quality of life, and blood pressure, improved. In children with primary snoring or mild OSAS, according to their PSG per conventional norms but with an SPS of >0.25, after other causes for sleepiness are ruled out, treatment with montelukast, nasal steroids, or even adenotonsillectomy could be justified. Conversely, two children with the same PSG measures but differing in SPS values would potentially serve to indicate that the one with the higher SPS would be more likely to benefit from treatment or merit more expedited access to treatment in light of the patient’s increased susceptibility.

However, it should be noted that although PSG clustering and machine learning are novel, potent tools for the diagnosis of OSAS, the conclusions of the respective studies are limited by the small sample sizes, lack of external validation, and heterogeneity in PSG scoring.

In children, the available literature suggests good to excellent test-retest reliability for respiratory PSG parameters [17,18], even though such might not be the case in adults [19]. There have been concerns that the first-night effect, characterized by decreased total sleep time, lower sleep efficiencies, reduction in REM sleep, and longer REM latencies due to sleeping in the unfamiliar environment of a sleep laboratory, may impact the results, and indeed differences in sleep architecture have been found. However, only minor differences in respiratory parameters were identified, and few children would have been clinically misclassified [3]. Clearly, as obstructive SDB is often more apparent or aggravated in REM sleep, care should be taken to check that there is sufficient REM sleep and total sleep time when reporting the PSG interpretation. Furthermore, should the parental report be that the night in the sleep laboratory was discrepant from a typical night’s sleep at home, this should be taken into consideration when interpreting the PSG findings.

## 2. Phenotypes and Endotypes

In medicine, the term “endotype” corresponds to a subtype of a disease, which is characterized by a distinct functional or pathobiological mechanism. In contrast, a “phenotype” involves any observable characteristic or trait of a disease without referring to a specific pathogenetic mechanism. Recognizing the underlying endotype may facilitate the application of targeted personalized interventions that will allow amelioration of clinical manifestations comprising the respective OSAS phenotype [20,21].

### 2.1. Endotypes in Pediatric OSAS (Figure 1)

In otherwise healthy children with OSAS-related symptoms and/or morbidity (Obstructive sleep apnea syndrome-OSAS, upper airway narrowing related to adenotonsillar enlargement is one of the most frequent underlying endotypes [1]. During the first eight years of life, nasopharyngeal lymphoid tissues grow proportionately to the size of the airway [22]. However, due to frequent exposure to respiratory viruses as well as other environmental pollutants and contaminants, the upper airway lymphadenoid tissues grow disproportionately to the size of the underlying airway, restricting the upper airway lumen to a variable degree [16,23,24,25,26,27,28,29]. After the age of eight, enlargement of the adenoid and palatine tonsils resolves in children without symptoms of sleep-disordered breathing such as snoring but persists in otherwise healthy children with snoring [30]. Therefore, the size of the nasopharyngeal and oropharyngeal airway lumen remains restricted in children with snoring, regardless of age [30]. Subtle craniofacial features in children, such as a retrusive chin or steep mandibular plane, which are not part of a major craniofacial malformation, may or may not contribute to increased upper airway resistance [31]. In addition, poor mandibular growth in the context of mouth breathing and the presence of allergic rhinitis may also favor the emergence of snoring and ultimately OSAS [32,33,34].

**Figure 1:**
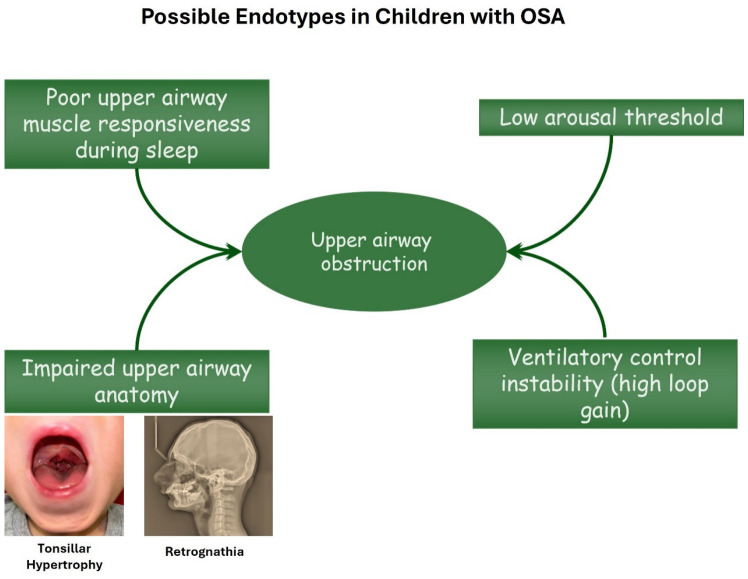
Schematic diagram illustrates the various possible endotypes underlying pediatric OSAS. Impaired upper airway anatomy resulting from adenotonsillar hypertrophy and/or retrognathia in combination with insufficient upper airway muscle responsiveness during sleep and a high loop gain (especially in overweight children) contribute significantly to the pathogenesis of OSAS.

If the degree of upper airway obstruction during sleep is appreciable as reflected by PSG parameters and if the airway obstruction is accompanied by phenotypical manifestations reflecting adverse health consequences and/or impaired quality of life, AT will frequently result in appreciable clinical and polysomnographic improvements, although not necessarily in complete OSAS symptom resolution [35,36,37].

Furthermore, accumulated evidence reveals that tonsillar T and B lymphocytes from children with OSAS overexpress leukotriene C4 synthase, which promotes the production of cysteinyl leukotrienes [38]. In addition, type-1 and type-2 cysteinyl leukotriene receptors are expressed by B lymphocytes in the tonsillar mantle zones and by T lymphocytes located in the extrafollicular areas, and it is also known that addition of the cysteinyl leukotriene D4 to a culture of adenoidal or tonsillar cells induces a proliferative response [39]. Thus, overproduction of cysteinyl leukotrienes in the pharyngeal lymphoid tissue of otherwise healthy children with enlarged adenoid and palatine tonsils and clinical manifestations of OSAS probably defines one of the most prevalent underlying endotypes. Treatment of children with adenotonsillar hypertrophy and mild OSAS with montelukast, an inhibitor of type 1 cysteinyl leukotriene receptors, has been used as a targeted intervention for this specific endotype, and it is usually accompanied by a reduction in the size of the adenoidal tissue and a decrease in the severity of intermittent upper airway obstruction during sleep. Similar results occur when the intervention consists of topical intranasal corticosteroids [40,41,42]. However, not all children with adenotonsillar hypertrophy snore or have OSAS, and tonsillar size alone does not predict OSAS severity [43,44].

In general, when the upper airway of children without OSAS is exposed to progressively more negative intraluminal pressure via an oronasal mask, there is essentially no change in the maximum inspiratory flow achieved within a wide range of negative pressures. These features reflect the robust protection of upper airway potency in children via neuromuscular mechanisms [45]. In contrast, in children with OSAS, maximum inspiratory flow decreases rapidly within a few cmH_2_O drop in intraluminal pressure, reflecting a tendency of their upper airway to collapse [46]. The fact that adenotonsillar hypertrophy does not necessarily predict the presence of OSAS implies that in a subgroup of children with pharyngeal lymphoid tissue hypertrophy and increased airflow resistance, the muscular part of the upper airway (30 pairs of muscles) most likely does not collapse on inspiration under the influence of the highly subatmospheric intraluminal pressure [47]. Therefore, neuromuscular dysfunction of the upper airway dilatory muscles is another OSA endotype in children [47,48,49,50].

Upper airway obstruction may be accompanied by hypoventilation and hypercapnia, which in normal children promotes neuromotor activation of the pharyngeal dilatory muscles and an increase in the maximum inspiratory flow. Nevertheless, this protective response of the respiratory center for the upper airway is not evident in a subgroup of children with OSAS, probably corresponding to an additional OSAS endotype (response to hypercapnia) [46].

Emerging phenotypes: Additionally, the relationship between CO_2_ production and alveolar minute ventilation is regulated by a closed-loop feedback system. The magnitude of the ventilatory response to hypercapnia accompanying an apnea event represents the “loop gain” of the ventilatory control system involving the respiratory center, upper airway, respiratory muscles, lungs, and circulation [51]. Factors decreasing the functional residual capacity, such as obesity, supine position, parenchymal lung disease, and neuromuscular disorders, amplify the effect of increased ventilation on alveolar pCO_2_ [52]. Hence, a small increase in ventilation may lead to a large decrease in alveolar/arterial pCO_2_ even below the apnea threshold, i.e., the pCO_2_ value below which an apnea occurs, aiming to reverse hypocapnia leading to respiratory instability [50,53]. Such a phenomenon (high loop gain) has been noted among subgroups of children who are overweight or who have a history of premature birth and may represent another OSAS endotype, but without immediate implications for a personalized treatment approach [49,50,54,55].

Finally, only a portion of obstructive apneas and hypopneas are accompanied by arousals in children with OSAS [56]. The term “respiratory arousal threshold” refers to the magnitude of mechanical effort or central respiratory drive that is required to arouse an individual with partial or complete collapse of the upper airway. A low respiratory arousal threshold is considered to be associated with respiratory instability due to an abrupt increase in the respiratory drive that is elicited by transition to wakefulness, a drop in arterial pCO_2_ below the apnea threshold, and recurrence of the apnea event. Hence, a “low respiratory arousal threshold” is considered an additional endotype in adult OSAS, which has not been confirmed in children [50,56,57].

### 2.2. Spectrum of Phenotypic Variation (See Table 1 and Figure 2)

In spite of these efforts, the correlation between PSG parameters and their associations with phenotypes of morbidity, the best studied is the AHI, and the severity of adverse consequences such as cardiovascular, metabolic, and CNS morbidity, is still suboptimal and clearly not readily applicable to the individual patient. It has now been conclusively demonstrated that the degree of overlap between disease severity and the frequency of morbidity manifestations is relatively modest. As such, when large cohorts of patients with varying degrees of sleep-disordered breathing severity are examined, a linear correlation emerges between AHI and the particular end-organ adverse consequence of choice. However, the variance of such a relationship is excessively large and allows for the fact that not all children with OSAS, even when severe, manifest end-organ involvement, whilst conversely, a proportion of children with primary snoring (i.e., “without disease”, despite the normal sleep study parameters, already present evidence suggestive of adverse sequelae [58]. Recent advances in the interpretation of PSG data using machine learning, EEG microstructure analysis, and hypoxic burden metrics could hopefully complement traditional PSG scoring and provide pathogenetic links between the severity of upper airway obstruction and end-organ morbidity.

**Table 1:** Factors that potentially impact end-organ morbidity.

	Factors That Potentially Impact End-Organ Morbidity
**PHENOTYPES-** **PSG PARAMETERS**	**Severity of OSAS** as reflected in parameters such as: AHI Hypoxic burden Sleep fragmentation Cycling alternating pattern Sleep spindles Functional MRI changes
	**Obesity**
	**Age, Pubertal status**
	**Ethnicity**
	**Concomitant medical co-morbidities**, e.g.,: Prader–Willi syndrome Trisomy 21 Sickle cell disease Neuromuscular diseases Epilepsy
**BIOMARKERS-** **OUTCOMES**	**Genetic polymorphisms**, e.g.,: NADPH oxidase p22 subunit gene polymorphisms (decreased risk of neurocognitive morbidity) Apolipoprotein E (APOE) ε4 (increased risk of neurocognitive morbidity) Genetic polymorphisms in nitric oxide synthase and endothelin gene families (endothelial dysfunction) Epigenetic alterations in the endothelial nitric oxide synthase gene (endothelial dysfunction) TNF-α gene polymorphisms (excessive daytime sleepiness)
	**Magnitude of underlying inflammatory response elicited**, e.g.,: Increased CRP levels increased neurocognitive morbidity) Decreased IGF-1 levels (increased neurocognitive morbidity) T regulatory lymphocytes (vascular manifestations)
	**Endothelial progenitor cells and other stem cells** (inability to reverse vascular injury)
	**Environmental factors (pollution), nutrition, lifestyle**

**Figure 2:**
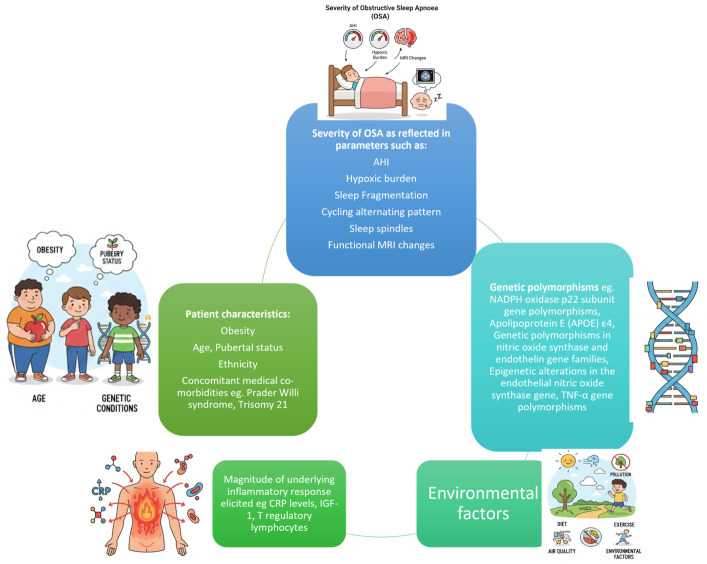
Factors that potentially impact end-organ morbidity. A high hypoxic burden, along with fragmented sleep related to OSAS in a genetically predisposed child exposed to adverse environmental factors such as pollution and an unhealthy diet, may trigger a profound inflammatory cascade, increasing the likelihood of developing end-organ morbidity.

#### 2.2.1. Neurocognitive and Behavioral Consequences

The potential variability between PSG scores and clinical outcomes is typified by what is seen in neurocognitive and behavioral consequences. Some children with severe OSAS do not appear to experience any morbidity in these functional domains, whereas some children with relatively mild OSAS or even with primary snoring exhibit behavioral problems that can frequently be (mis)construed as attention deficit and hyperactivity disorder. In a large community cohort of young school-aged children, as a group, those with moderate-to-severe OSAS showed an increased risk for neurocognitive impairments compared with controls, children with primary snoring, and children with mild OSAS [59]. However, there was significant overlap between the groups. Furthermore, children with primary snoring performed more poorly on the NEPSY Visual Attention subtest than children with mild or moderate/severe OSAS.

Indeed, PSG indices such as AHI, arousal index, percentage of time asleep, oxygen desaturation of hemoglobin nadir, mean SpO_2_, time spent with SpO_2_ < 90%, maximum CO_2_, and percentage of total sleep time with hypercapnia (CO_2_ > 50 mmHg) have not been found to be predictive of neurocognitive dysfunction [60,61]. Notwithstanding, a recent study proposed a novel hypoxic burden biomarker that appeared to facilitate better prediction of cognitive dysfunction in children with OSAS [62]. This is an important effort to delineate one or more PSG-derived severity markers that collectively may identify enhanced risk for cognitive problems in affected children [63].

A possible explanation for these weak associations is that some at-risk children may not be recognized by the current diagnostic methods. This has led to the study of alternative indices such as cyclic alternating pattern (CAP) analysis and analysis of spindle activity. CAP analysis is a method to assess the microstructure of sleep. In the AASM scoring rules, EEG arousals are defined as abrupt changes in the EEG of a minimum duration of 3 s, and changes in the shorter duration are not taken into consideration. CAP analysis captures dynamic changes in EEG amplitude and frequency that recur periodically in NREM sleep [64]. Such sequences of recurring activation phases represent periods of high neural excitability with an intervening background interval and provide insights into the fragmentation of NREM sleep that otherwise would not be possible with traditional sleep staging [65]. Children with OSAS have been shown to have a lower CAP rate and reduced entropy compared with controls [66]. A higher number of A1 phases per hour of sleep was significantly associated with worse behavioral functioning and lower quality of life in children with moderate OSA [67].

Sleep spindles are bursts of 10–16 Hz EEG activity, typically between 0.5 and 2 s in duration. Sleep spindle activity has been associated with memory consolidation, IQ, and cognitive performance. A pilot study has shown that children with mild OSAS demonstrate a different pattern of sleep spindle activity compared with controls, and the spindle index correlated with Verbal Comprehension Index, Working Memory Index, Processing Speed Index, and total IQ [68]. Children with primary snoring have also been shown to have reduced spindle activity compared with controls [69]. These changes may be an indicator of subtle sleep disruption and a factor in disease-related consequences.

Another explanation for the variance is the individual variability in neuroplasticity and the adoption of differential compensatory mechanisms, which are influenced by genetic and environmental factors. Functional MRI imaging data of the brain have shown that children with OSAS require greater neural recruitment of regions of the brain involved in cognitive control, conflict monitoring, and attentional allocation in order to perform at the same level as those without OSAS [70]. Several regions of cortical thinning as well as changes in gray and white matter regional brain volumes have also been described in children with OSAS, though once again the severity of OSAS did not necessarily correlate with the extent of these alterations [71,72]. Substantial efforts with advanced brain MRI techniques have yet to enable improved prediction of morbidity, but are shedding some light on the involvement of white matter tracts, reconfiguration of resting-state brain activity, and overall topology and functional networks [73,74,75,76,77,78].

Inflammatory factors appear to explain some of the individual variability seen. Children with OSAS who have neurocognitive dysfunction have been reported to have higher plasma CRP levels compared to children with OSAS without [79]. Thus, the magnitude of the underlying inflammatory response elicited by OSAS is likely a factor determining the increased risk for neurocognitive deficits. Furthermore, circulating IGF-1 levels have been shown to be decreased in children with OSA with neurocognitive dysfunction compared to those without [80]. IGF-1 is thought to play a neuroprotective role in hypoxia [81] and is known to stimulate the production of the anti-inflammatory cytokine IL-10 in human T cells, which has been shown to be decreased in pediatric OSAS [82].

Genetic polymorphisms likely explain at least some of the variability in neurocognitive and behavioral morbidity. NADPH oxidase p22 subunit gene polymorphisms have been identified as potential modifiers of risk for neurocognitive deficits in children with the rs4673 SNP of the NADPH oxidase p22 subunit has reduced NADPH oxidase activity, and consequently reduced oxidative stress, and was less likely to manifest neurocognitive morbidity [83]. Conversely, the Apolipoprotein E (APOE) ε4 allele has been shown to be more frequent in children with OSAS, particularly in those with neurocognitive dysfunction, suggesting it may be associated with an increased risk for OSAS and its associated deleterious neurocognitive consequences [84].

#### 2.2.2. Cardiovascular Morbidity

The significant overlap between children with OSAS and those with neurocognitive deficits and endothelial dysfunction suggests these morbid consequences may share similar pathogenetic mechanisms [85]. The severity of endothelial dysfunction is greater in obese children than in non-obese children who have OSAS [86]. One of the factors contributing to variance in endothelial functional phenotype may reside in the ability to recruit endothelial repair mechanisms. Despite similar OSAS severity, circulating endothelial progenitor cell (EPC) counts and plasma stromal cell-derived factor-1 (SDF-1) levels have been shown to be significantly lower in those OSAS children with endothelial dysfunction, compared with OSAS children who have normal endothelial function or two controls [87]. EPCs have the potential to reverse vascular injury, and their numbers are inversely correlated with the severity of endothelial dysfunction.

Similarly, T lymphocytes may also contribute to the vascular manifestations of pediatric OSAS. Increased DNA methylation of the FOXP3 gene, the transcription factor that regulates T regulatory cells (T regs), has been reported in pediatric OSAS and likely induces reduced activity of Treg lymphocytes, thereby enabling a pro-inflammatory state [88]. Indeed, T regs usually function as immunomodulators to mitigate and extinguish pro-inflammatory responses and have been shown to inhibit the development and progression of atherosclerosis, with an inverse correlation being observed between the percentage of Tregs and the severity of endothelial dysfunction [89].

Endothelial dysfunction typically improves after treatment of the OSAS. However, in a subset of patients with a strong family history of cardiovascular disease, it can remain abnormal despite objective resolution of the OSAS [90]. Thus, not only is there phenotypic variation in the degree of cardiovascular morbidity in relation to disease severity, but there is also variation in the degree of morbidity resolution.

Genetic polymorphisms in nitric oxide synthase and endothelin gene families, as well as epigenetic alterations in the endothelial nitric oxide synthase (eNOS) gene, have also been identified as potential modifiers of endothelial dysfunction in the context of pediatric OSAS [91,92]. However, a comprehensive whole-genome assessment of genetic variants associated with phenotypic heterogeneity is lacking and is clearly needed if we wish to improve the predictability of phenotypes and personalize clinical care. In recent years, the exploration of microRNAs as potential biomarkers and also as mechanistic effectors of end-organ morbidity has emerged in both adults [93,94,95] and in children [96].

Nevertheless, it should be noted that currently the reported associations between biomarkers and OSAS-induced end-organ morbidity remain exploratory, and they are not yet ready for routine clinical use. Large cohort multicenter interventional studies are lacking at this stage and will be critically needed to improve upon the implementation of the highly desirable goal of personalized medicine in pediatric OSAS.

#### 2.2.3. Metabolic Morbidity

The associations between pediatric OSAS and insulin resistance and dyslipidemia are likely moderated by the presence of obesity, but also by factors such as age, ethnicity, and pubertal status. In post-pubertal adolescents, a strong association has been demonstrated between OSAS and the metabolic syndrome [97], but in pre-pubertal children, the association with decreased insulin sensitivity becomes apparent only when obesity is concurrently present [98,99]. Whilst obesity appears to be the primary driver of most associations between OSAS and metabolic measures, sleep fragmentation has been shown to be positively associated with insulin resistance measures [100]. There appears to be a complex interplay between obesity and OSAS, both being conditions where there is low-grade inflammation and overlapping cardiometabolic morbidities.

Another example of the interaction between obesity and OSAS is in the manifestation of excessive daytime sleepiness (EDS). Unlike adults suffering from OSAS, EDS is generally not as common a symptom in pediatric OSAS. However, it is more prominent in obese patients and those with more severe OSAS [101]. An association between elevated plasma TNF-α levels and EDS has been described, albeit inconsistently [102,103]. The degree of obesity and variations in the population distribution of TNF-α gene polymorphisms, as well as other genes encoding for cytokines mediating sleep, may contribute to this variability [104,105,106,107,108,109].

### 2.3. Obstructive Hypoventilation

There is less research published on obstructive hypoventilation in children. This is more commonly seen in children with co-morbidities such as severe obesity or in children affected by syndromic disorders. For example, hypoventilation disproportionate to OSAS severity has been described in children with Prader–Willi syndrome [110]. Children with Down syndrome have also been shown to have increased mean CO_2_ during sleep, regardless of the presence of OSAS and its severity [111]. In obese children, the degree of hypoventilation does not appear to correlate well with symptoms, OAHI, or BMI, leading to the postulation that compensation for the increased load on the respiratory system shows significant interindividual differences. Often, the presence of hypoventilation results from the multifactorial combination of anatomical features such as adenotonsillar hypertrophy, midface hypoplasia, micrognathia, hypotonia, and/or ventilatory control alterations that result in hypoventilation. Certainly, children with Prader–Willi syndrome are known to have blunted hypoxic and hypercapnic ventilatory responses and absent peripheral chemoreceptor sensitivity [112,113].

## 3. Opportunities for Personalized Interventions

Adenotonsillectomy (AT) is the recommended first-line management for pediatric OSAS [3]. However, up to 40% of children may have residual OSAS, the risk of which is even higher in those with severe OSAS, the obese, and patients with medical co-morbidities or genetic conditions, such as Down syndrome and craniofacial syndromes [37].

Accurate assessment of the child’s upper airway creates the opportunity for personalized intervention. This can be achieved via upper airway endoscopy or dynamic upper airway imaging. Drug-induced sleep endoscopy (DISE) is currently the most commonly used tool for upper airway evaluation, and this review will use it as an example of how upper airway assessment can guide individualized treatment.

### Drug-Induced Sleep Endoscopy

DISE is a technique that entails evaluation of the upper airway using a flexible endoscope while patients are in pharmacologically induced sedation. The endoscope is passed through the nares to examine the nasopharynx, oropharynx, and larynx, in order to identify the sites of obstruction and guide subsequent surgical treatment. It is particularly useful as part of the assessment of patients with persistent OSAS after AT [114] as it guides surgical decision-making, enabling the choice of subsequent interventions. These include repeat adenoidectomy, supraglottoplasty, lingual tonsillectomy, nasal surgery such as inferior turbinate submucosal resection, and uvulopalatoplasty, which may be required to enable subsequent improvement in OSAS [115,116,117]. An example is the recent meta-analysis on DISE-directed tongue surgery that found that DISE-directed tongue surgery in children with persistent OSAS after AT was effective, leading to reductions in AHI from 9.5 ± 12.1 to 4.2 ± 6.9 events/h TST [118].

The indications for DISE are expanding and now also include surgically naïve patients at high risk for persistent OSAS, e.g., those who are obese, have Down syndrome, craniofacial anomalies, etc. Similarly, DISE is also being increasingly used in children with OSA with small tonsils and adenoids, children with symptoms suggestive of sleep-state dependent laryngomalacia, and as part of an evaluation to determine whether hypoglossal nerve stimulation might be a therapeutic option [119,120]. This last indication is of particular recent interest, as hypoglossal nerve stimulation is one of the more novel treatment options that have emerged. The device stimulates the hypoglossal nerve, with resultant tongue protrusion and airway opening on inspiration during sleep. It has been shown to be effective in adults with moderate-to-severe OSAS who are intolerant of positive airway pressure (PAP) therapy. Theoretically, hypoglossal nerve stimulation may be particularly suitable for patients with Down syndrome because it can augment neuromuscular airway tone and potentially reduce obstruction at the level of the tongue base, which is one of the more common sites of obstruction in these children. Indeed, a trial of 42 adolescents with Down syndrome and persistent severe OSAS despite AT who were not tolerant of PAP therapy showed significant improvement in AHI and quality of life outcomes, with acceptable complication rates. The most common complications consisted of temporary tongue or oral discomfort [121]. DISE is needed in the pre-evaluation process for hypoglossal stimulation, as patients who have concentric collapse at the level of the velum are unfortunately ineligible for this procedure.

DISE likely has an even more prominent role to play in younger children, enabling stratification and giving an insight into pathogenesis. Infants and young children < 2 years represent a unique subgroup when it comes to OSAS, as OSAS is often a multifactorial disorder that requires objective assessment and treatment of all underlying abnormalities that contribute to the upper airway obstruction during sleep [122]. When Blancke et al. performed DISE on 100 patients aged ≤ 2 years, adenoid and/or tonsillar hypertrophy was found in 31% of the cohort, 21% had laryngomalacia, and 26% had multilevel collapse [123]. Other conditions identified were subglottic stenosis (2%), glottis oedema (1%), tracheomalacia (2%), intubation trauma 1%, palatal collapse (1%), epiglottic collapse 1%, and oropharyngeal collapse 1%. A change in pattern was noted with age, whereby laryngomalacia was highly prevalent in infants < 6 months compared with none in children < 1 year old. Conversely, adenotonsillar hypertrophy was not seen in infants < 6 months but was the most common finding in those aged 1.5–2 years. In this study, treatment was given based on PSG data, DISE findings, and shared decision-making between the child’s parents and the multidisciplinary team. About 75% underwent surgical treatment. Supraglottoplasty was performed in 18/21 of the patients with laryngomalacia and resulted in significant improvements in the AHI. The remaining 3/21, one of whom had pharyngo-laryngomalacia and 2 of whom had type 3 laryngomalacia with a collapse of the epiglottis against the posterior pharyngeal wall, were treated with CPAP. Similarly, 88% of the children with multilevel collapse were treated with adenotonsillar surgery with significant improvement in the AHI [123].

**Figure 3:**
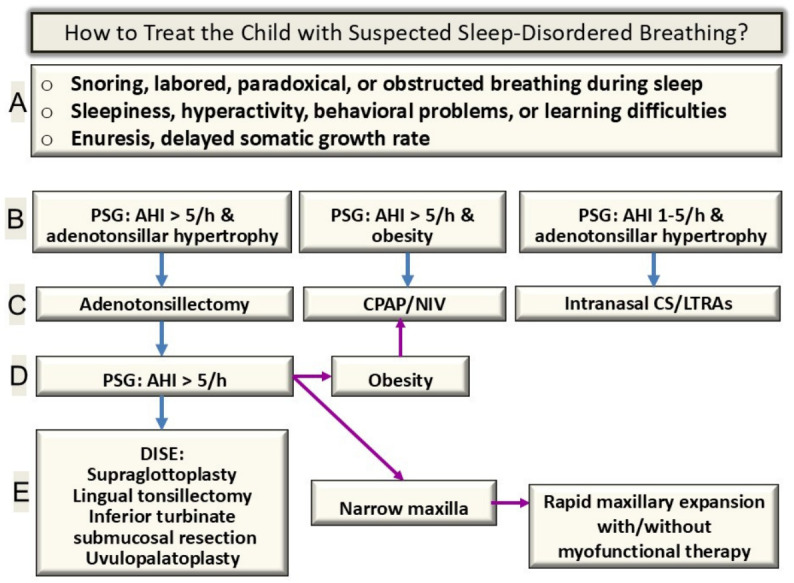
Combinations of endotypes/phenotypes and proposed treatment interventions; starting at A and progressively advancing to E as needed.

Figure 3 summarizes potential combinations of endotypes/phenotypes and proposed treatment interventions.

## 4. Clinical Implications

As can be seen, the substantial phenotypic variability is indicative of the complex interplay of intrinsic anatomical, functional, genetic, and environmental determinants, along with the presence of other underlying co-morbidities, which collectively determine the susceptibility to and severity of OSAS morbidity. Whilst information obtained from a PSG is a helpful steer, and there is general agreement that a child with moderate-to-severe OSAS should receive treatment, there may be times when treatment may be considered in patients irrespective of the PSG measures as currently derived. Examples include patients with primary snoring who already display deleterious cardiovascular or neurocognitive sequelae, or children with complex underlying conditions who may be at particular risk of morbidity. Objective evaluation of the upper airway, for example, using DISE, can help personalize treatment. Some initial directions and proposals regarding precision approaches in adult OSAS have emerged, and similar considerations should be explored in the pediatric realm, particularly considering the relatively higher risk imposed by a decision to implement surgical treatment such as AT [124,125,126].

Although CPAP is the main current treatment in children and adolescents with body mass index values in the obesity range, for those unable or not eager to use CPAP, bariatric surgery and the recent introduction of Glucagon-Like-Peptide-1 receptor agonists have been associated with promising results in adults [127]. Moreover, myofunctional therapy involves a series of oropharyngeal exercises aiming at improving muscle tone in order to maintain airway patency [128]. Studies of low methodological quality indicate that myofunctional therapy may decrease OSAS severity and associated symptoms.

Maxillary restriction and retrognathia/malocclusion represent a distinct group of abnormalities predisposing otherwise healthy children to OSAS. Rapid maxillary expansion has been used successfully in children with OSAS and maxillary constriction, while custom-made oral appliances have offered promising results in cases of OSAS with retrognathia or malocclusion [129,130].

Table 2 summarizes current actionable and investigational treatment approaches for the management of OSAS in children. Overall, the use of investigational treatment approaches is currently limited in the context of small sample sizes, lack of external validation, heterogeneity of PSG scoring, and absence of prospective interventional trials showing improved outcomes.

## 5. Conclusions

Obstructive SDB encompasses a wide and complex spectrum of disease. As the AASM highlighted, the “gold standard” for the diagnosis of sleep-related breathing disorders in children is not PSG alone, but rather the skillful integration of clinical and PSG findings by a knowledgeable and experienced sleep specialist. It is crucial that management should not only be guided by PSG results in isolation, but that the overall clinical picture, including information from the history on symptomatology, examination findings, presence of risk factors, and associated disease morbidity, needs to be systematically evaluated. This information should then be incorporated into the formulation of an individualized treatment plan, the potential for personalized medicine resting on the generation of a precise diagnosis with patient-specific targeted interventions.

## Figures and Tables

**Table 2:** Actionable and investigational approaches for the management of OSAS in children.

Actionable Approaches to OSAS Management	Investigational Approaches to OSAS Management
**IMPAIRED UPPER AIRWAY** **ANATOMY** Adenotonsillectomy for adenotonsillar hypertrophy Intranasal corticosteroids and montelukast for adenoidal hypertrophy accompanied by mild OSAS Inferior turbinate submucosal resection for nasal turbinate mucosal hypertrophy Supraglottoplasty for laryngomalacia Lingual tonsillectomy for lingual tonsil hypertrophy Rapid maxillary expansion for maxillary constriction and custom-made oral appliances for retrognathia or malocclusion	
**POOR UPPER AIRWAY MUSCLE** **RESPONSIVENESS DURING SLEEP** CPAP for oropharyngeal collapse, especially in cases of obesity	**For patients unable or not eager to use CPAP:** Bariatric surgery Glucagon-Like-Peptide-1 receptor agonists Hypoglossal nerve stimulation Myofunctional therapy
**IMPAIRED UPPER AIRWAY** **ANATOMY AND POOR UPPER AIRWAY MUSCLE RESPONSIVENESS** Uvulopalatopharyngoplasty for patients with a narrow oropharyngeal airspace who cannot tolerate CPAP	

## Data Availability

No new data were created or analyzed in this study.

## Abbreviations

AASM	American Academy of Sleep Medicine
AHI	Apnea-hypopnea index
APOE	Apolipoprotein E
ARtotI	Arousal index
AT	Adenotonsillectomy
CAP	Cyclic alternating pattern
CHAT	Childhood Adenotonsillectomy Trial
CPAP	Continuous positive airway pressure
DISE	Drug-induced sleep endoscopy
EDS	Excessive daytime sleepiness
eNOS	Endothelial nitric oxide synthase
EPC	Endothelial progenitor cell
MRI	Magnetic resonance imaging
NIV	Noninvasive ventilation
OSAS	Obstructive sleep apnea syndrome
PAP	Positive airway pressure
PSG	Polysomnography
RAI	Respiratory arousal index
SAI	Spontaneous arousal index
SDB	Sleep-disordered breathing
SDF-1	stromal cell-derived factor-1
SPS	Sleep Pressure Score
TST	Total sleep time
UARS	Upper airway resistance syndrome
